# Expression of tryptophan 2,3-dioxygenase in mature granule cells of the adult mouse dentate gyrus

**DOI:** 10.1186/1756-6606-3-26

**Published:** 2010-09-05

**Authors:** Koji Ohira,, Hideo Hagihara,, Keiko Toyama,, Keizo Takao, Masaaki Kanai, Hiroshi Funakoshi, Toshikazu Nakamura, Tsuyoshi Miyakawa

**Affiliations:** 1 Division of Systems Medical Science, Institute for Comprehensive Medical Science, Fujita Health University, Toyoake 470-1192, Japan; 2 Japan Science and Technology Agency (JST), Core Research for Evolutional Science and Technology (CREST), Kawaguchi 332-0012, Japan; 3 Genetic Engineering and Functional Genomics Group, Frontier Technology Center, Graduate School of Medicine, Kyoto University, Kyoto 606-8501, Japan; 4 Center for Genetic Analysis of Behavior, National Institute for Physiological Sciences, Myodaiji, Okazaki, 444-8585, Japan; 5 Department of Biochemistry and Molecular Biology, Division of Molecular Regenerative Medicine, Osaka University Graduate School of Medicine, Osaka 565-0871, Japan; 6 Kringle Pharma Joint Research Division for Regenerative Drug Discovery, Center for Advanced Medicine, Osaka University, Osaka 565-0871, Japan

## Abstract

New granule cells are continuously generated in the dentate gyrus of the adult hippocampus. During granule cell maturation, the mechanisms that differentiate new cells not only describe the degree of cell differentiation, but also crucially regulate the progression of cell differentiation. Here, we describe a gene, tryptophan 2,3-dioxygenase (TDO), whose expression distinguishes stem cells from more differentiated cells among the granule cells of the adult mouse dentate gyrus. The use of markers for proliferation, neural progenitors, and immature and mature granule cells indicated that TDO was expressed in mature cells and in some immature cells. In mice heterozygous for the alpha-isoform of calcium/calmodulin-dependent protein kinase II, in which dentate gyrus granule cells fail to mature normally, TDO immunoreactivity was substantially downregulated in the dentate gyrus granule cells. Moreover, a 5-bromo-2'-deoxyuridine labeling experiment revealed that new neurons began to express TDO between 2 and 4 wk after the neurons were generated, when the axons and dendrites of the granule cells developed and synaptogenesis occurred. These findings indicate that TDO might be required at a late-stage of granule cell development, such as during axonal and dendritic growth, synaptogenesis and its maturation.

## Background

Throughout life, new neurons are continuously generated in the subgranular zone of the hippocampal dentate gyrus (DG) and in the anterior subventricular zone of the lateral ventricles [1-3]. In the adult hippocampus, neural stem cells/progenitor cells and postmitotic granule cells are distributed in distinct locations [2-4]. Neural stem cells exist near the border between the hilus and the DG granule cell layer. Neuroblasts generated in the subgranular zone migrate radially a short distance into the granule cell layer and are integrated into the deepest portion of the granule cell layer, where they differentiate into granule cells, extending dendrites and axons and receiving synaptic inputs. During development, new granule cells express several marker proteins that depend on the level of cell differentiation [2,3]. Granule cell progenitors develop in three stages, namely type 1, 2, and 3 progenitors. Type 1 progenitors express nestin, glial fibrillary acidic protein (GFAP), brain lipid-binding protein, and sox2. Type 2 and 3 progenitors express Tbr2, doublecortin (DCX), and polysialic acid-neural cell adhesion molecule (PSA-NCAM). The latter two markers also exist at an early stage of postmitotic cell development [2,3]. These molecules play key roles in the development of granule cells [5], and may thus be involved in the proliferation of type 1 progenitor cells [6-8]. DCX, a microtubule binding protein, is transiently expressed during migration and outgrowth of neuroblast axons and dendrites [9]. Because DCX bundles and stabilizes microtubules, it may also have a role in developmental events, such as the reorganization of microtubules. PSA-NCAM seems to be involved in structural plasticity, such as cell migration and outgrowth of dendrites and axons [4,10], and its expression almost completely overlaps with that of DCX [2,3]. Thus, it is important to identify marker proteins and their functions in these cells.

In mice heterozygous for the alpha-isoform of calcium/calmodulin-dependent protein kinase II (CaMKIIα), almost all hippocampal granule cells fail to mature and remain in an immature state [11]. The behavioral abnormalities in these mice include a severe working memory deficit and an exaggerated infradian rhythm, which are similar to symptoms observed in patients with schizophrenia, bipolar mood disorder, and other psychiatric disorders [11]. Transcriptome analysis of the hippocampus of these mutants revealed significant changes in the expression of more than 2000 genes [11]. Further, among the 20 most downregulated genes in the CaMKIIα+/- hippocampus, the mRNA expression level of tryptophan 2,3-dioxygenase (TDO) was only 9.2% that of the control levels [11]. Based on the maturation failure of the granule cells of CaMKIIα+/- mice and the higher expression of *tdo* in the hippocampus compared with several regions of the central nervous system [12], it can be inferred that TDO might be expressed only after a certain stage of granule cell differentiation in the normal mouse.

TDO is a rate-limiting enzyme for the kynurenine pathway of tryptophan (Trp) metabolism [13], and Trp concentrations in the hippocampus of *Tdo*-/- mice are 20-fold higher than that in control mice [14]. In the hippocampus of *Tdo*-/- mice, levels of serotonin (5-HT), which is synthesized from Trp, are 2-fold higher than that in control mice [14]. In addition, adult neurogenesis is significantly upregulated in the hippocampus and anterior subventricular zone in *Tdo*-/- mice [14]. Taken together, these findings indicate that TDO might be a key molecule involved in regulating adult hippocampal neurogenesis.

In the present study, to further clarify the role of TDO, we determined the expression pattern of TDO in the granule cells of adult mouse hippocampus using single- and double-immunofluorescence microscopy. We further examined the onset of TDO expression using 5-bromo-2'-deoxyuridine (BrdU) immunohistochemistry.

## Results

### TDO expression in granule cells

**Figure 1:**
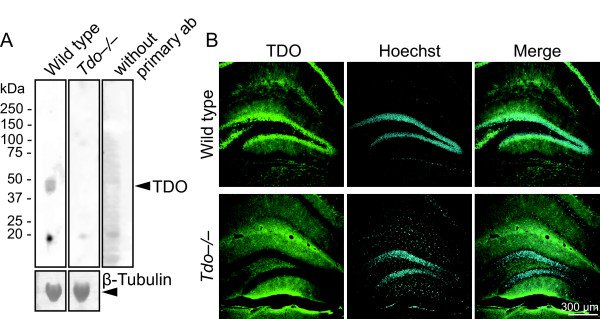
**Specificity of antibodies for tryptophan 2,3-dioxygenase (TDO) by Western blot (A) and immunofluorescent staining (B)**. **A: **The total protein (20 μg) of the dentate gyrus, extracted from the wild-type (lanes 1 and 3) and *Tdo*-/- mice (lane 2), was subjected to 4% to 12% gradient sodium dodecyl sulfate polyacrylamide gel electrophoresis, and transferred to polyvinylidene difluoride membranes. TDO was detected as described in the Methods section. Note that a 45-kDa band was detected with the anti-TDO antibody. The positions of molecular weight markers are shown on the left. Lane 3: negative control without the primary antibody. β-Tubulin is a positive control for the Western blot. **B: **The hippocampal sections of wild-type and *Tdo*-/- mice were stained with anti-TDO antibody. In sections of *Tdo*-/- mice, immunofluorescence signals were not detected in granule cells, interneurons (arrowhead), CA1, or CA3 cells, suggesting that the secondary antibody used in this study bound specifically to the primary antibody (mouse IgG). g, granule cell layer; h, hilus.

During the development of granule cells in the adult hippocampus, granule cell progenitors transiently express some marker proteins, GFAP for type 1 neural progenitors and DCX for types 2 and 3 progenitors [2,3]. Using these established marker proteins, we examined whether TDO was expressed in the progenitors. Marked reduction of TDO immunoreactivity in Western blotting and immunostaining of the hippocampus of *Tdo*-/- mice as compared with wild type mice showed the specificity of TDO immunostaining (Fig. 1).

**Figure 2:**
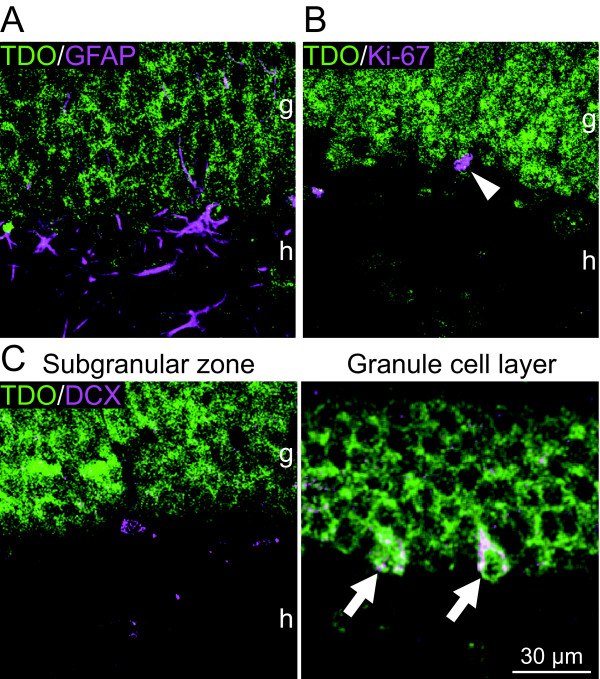
**TDO immunoreactivity in progenitor cells**. **A: **The immunoreactivity of a type I progenitor cell marker, glial fibrillary acidic protein (GFAP), was rarely observed in TDO-expressing cells. **B**: Cells double-labeled with Ki67 and TDO were scarcely observed (arrowhead). **C: **Co-expression of TDO with doublecortin (DCX). Few DCX-positive (+) cells in the subgranular zone, which comprises type 2 progenitor cells, contained TDO. In contrast, DCX+ cells that integrated into the granule cell layer, which comprises postmitotic immature neurons, co-expressed TDO (arrows). g, granule cell layer; h, hilus.

GFAP immunoreactivity was observed mainly in the subgranular zone, and was not co-labeled with TDO+ cell bodies (0 of 177 GFAP+ cells, n = 3 mice; Fig. 2A). GFAP immunoreactivity was also observed in the granule cell layer. Because astrocytes extend their processes into the granule cell layer, these GFAP-positive (+) processes appeared between TDO+ granule cells (Fig. 2A). Thus, we concluded that few type 1 progenitors express TDO.

All progenitors are proliferative and therefore contain the cell proliferation marker Ki-67, which is a nuclear protein expressed during all phases of the cell cycle except G_0 _[15]. Few Ki-67+/TDO+ granule cells were observed (0 of 101 Ki-67+ cells, n = 3 mice; Fig. 2B). The result of the DCX staining suggests that TDO expression starts at a late developmental stage, such as at the end stage of type 3 progenitors or immature cells, and might not be expressed in proliferating progenitors.

We further evaluated the coexpression of TDO in DCX+ cells. DCX is expressed not only in type 2 and 3 progenitors, but also in immature granule cells in the adult hippocampus [2,3,9]. The expression of DCX lasts for up to 4 wk after the generation of granule cells [9]. Type 2 and 3 progenitors are easily distinguished from immature cells in the adult DG by their distribution; namely progenitors are located in the subgranular zone and immature cells are located in the granule cell layer [4]. In the subgranular zone, the rate of TDO+ cells among DCX+ cells was low (Fig. 2C, left image), 10.2 ± 4.34% (mean ± SEM, n = 3 mice). On the other hand, in the granule cell layer, although the number of DCX+ cells was quite small, almost all DCX+ cells expressed TDO, 78.1 ± 6.64% (n = 3 mice; Fig. 2C, right panel). Thus, these results suggest that TDO expression gradually increased with the development of granule cells.

**Figure 3:**
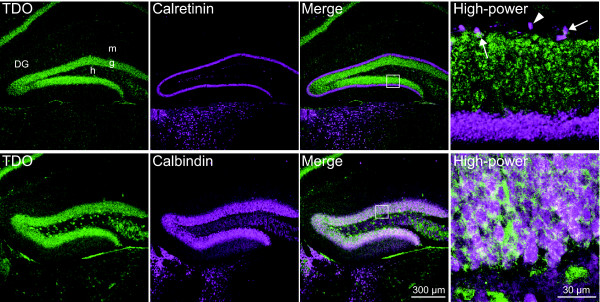
**TDO immunoreactivity in immature and mature granule cells**. **A: **Immature granule cells that were calretinin+ contained TDO. Arrows indicate examples of double labeling of calretinin with TDO. Arrowheads indicate cells labeling for calretinin only. **B: **Co-labeling of a mature granule cell marker, calbindin, with TDO. Note that almost all calbindin+ granule cells expressed TDO. Higher magnifications of the boxed-in areas in the merged images are displayed in the right-hand row. g, granule cell layer; h, hilus; m, molecular layer.

**Figure 4:**
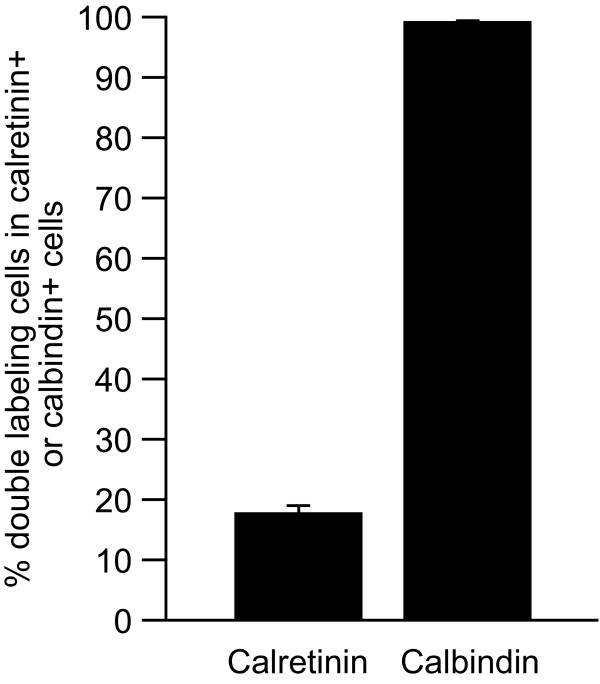
**Co-labeling rates of TDO expression in immature (calretinin+) or mature (calbindin+) granule cells**. The cell count data from Fig. 3 were quantified. Values are given as the means ± SEM of the analysis based on the results of four mice (total cell counts: calretinin, n = 1629; calbindin, n = 2354).

Immature granule cells can be distinguished from type 3 progenitors by the expression of calretinin and their location in the deepest granule cell layer [2,3,16]. On the other hand, mature granule cells express calbindin and are integrated into the granule cell layer [2,3,17]. Thus, it is capable of distinguishing immature granule cells from mature granule cells by the expression of the cell markers calretinin and calbindin. To evaluate the pattern of TDO expression within postmitotic granule cells, we performed immunofluorescence double-labeling for calretinin and calbindin with TDO. Calretinin+/TDO+ cells were detected in the subgranular zone and in the deepest granule cell layer (Fig. 3). Almost all calbindin+/TDO+ cells were distributed in the granule cell layer (Fig. 3). TDO+ cells comprised 284 of 1629 calretinin+ (17.7 ± 6.33%, n = 4 mice) and 2332 of 2354 calbindin+ cells (99.1 ± 0.787%, n = 4 mice; Fig. 4), suggesting that some immature granule cells, which might be at the late-stage of development, and mature cells express TDO.

**Figure 5:**
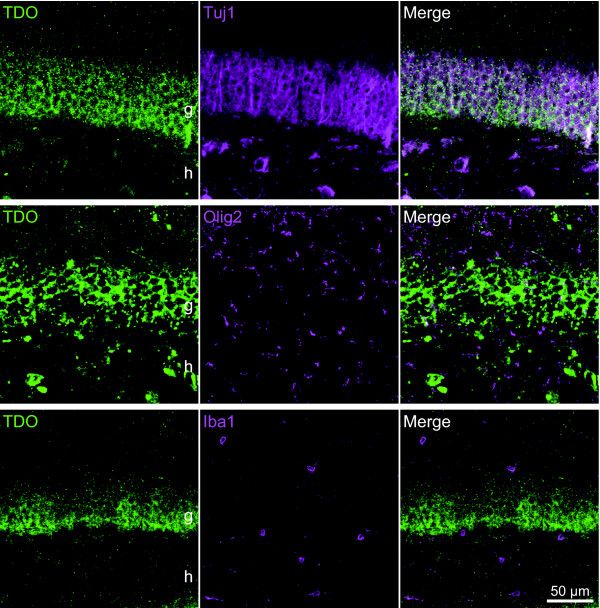
**Neuronal immunoreactivity of TDO in the dentate gyrus**. Immunofluorescent analysis revealed that TDO was expressed in neurons (Tuj1), but not in oligodendrocytes (Olig2) and microglias (Iba1). Almost all Tuj1+ neurons were immunoreactive for TDO. g, granule cell layer; h, hilus.

We determined if TDO+ cells were neurons or glial cells, using neuronal and glial cell markers. Almost all TDO+ cells were stained by Tuj1 antibody, which was reactive to neuronal class III beta-tubulin, but not by anti-Olig2, or anti-Iba1 antibodies and not mostly by anti-GFAP, (Figs. 2A and 5). This data suggest that TDO was expressed specifically in neurons in the adult mouse hippocampus under physiological conditions.

**Figure 6:**
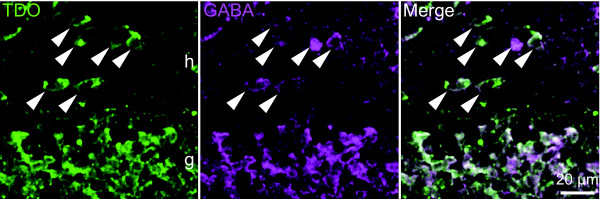
**Immunoreactivity of TDO in the dentate hilar interneurons**. TDO-expressing cells in the dentate hilus contained GABA. Arrowheads indicate TDO and GABA double-positive cells. g, granule cell layer; h, hilus.

In the subgranular zone and hilus, TDO+ cells were also observed, but these cells had larger cell bodies than the granule cells (Fig. 6). These TDO+ cells possessed GABA. Thus, the TDO+ cells in the subgranular zone and hilus are GABAergic interneurons, such as pyramidal basket cells and mossy cells, which are located in the hilus or at the border between the granule cell layer and the hilus [18].

**Figure 7:**
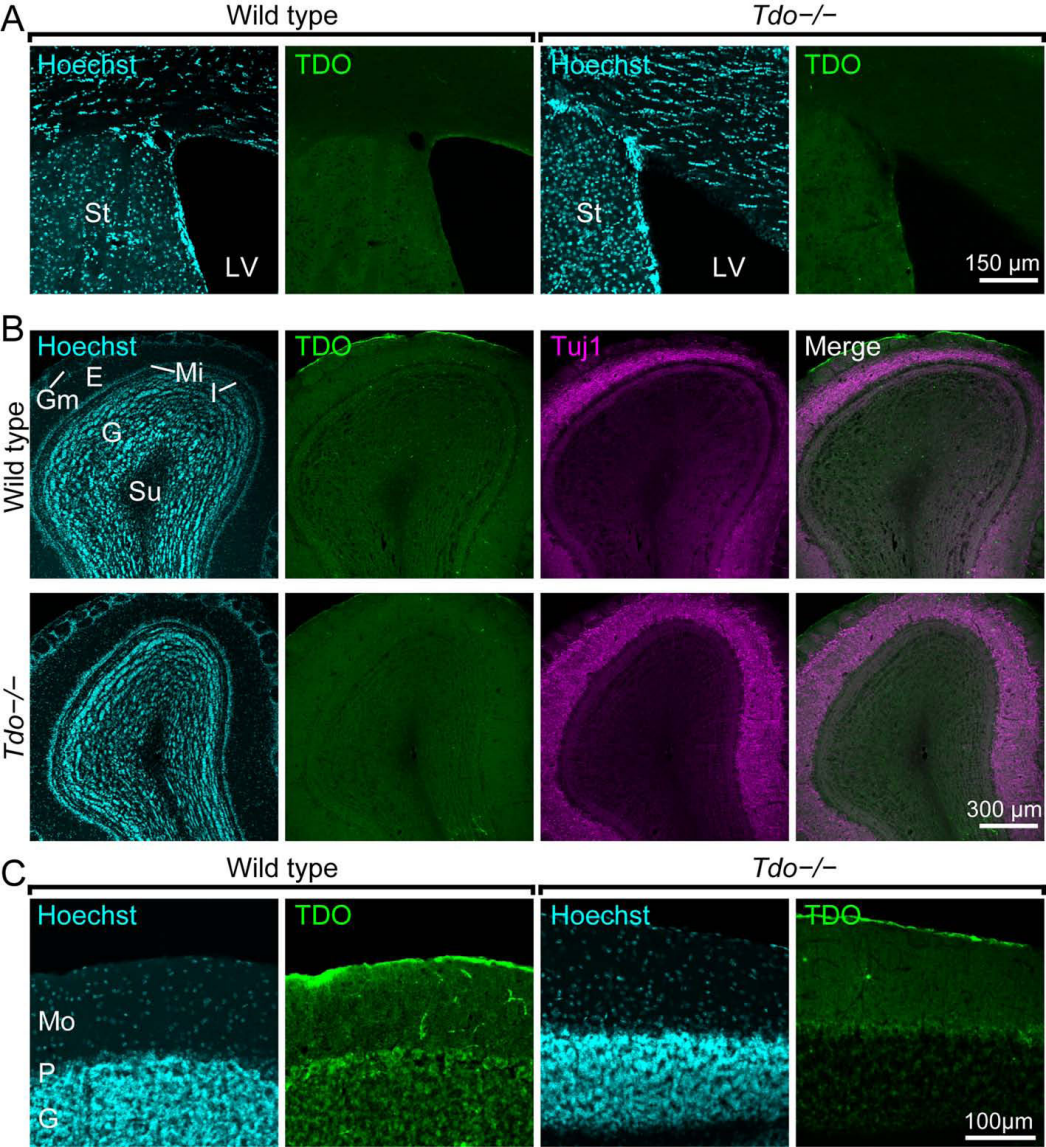
**Immunoreactivity of TDO in the SVZ, the olfactory bulb, and the cerebellum**. **A **and **B: **TDO was not stained in the SVZ (A) and the olfactory bulb (OB) (B). The immunoreactivities for TDO in wild type and *Tdo*-/- mice were background level. **C: **In the cerebellum, the immunoreactivity for TDO was observed in granule cells and Purkinje cells of wild type mice, but these positive structures disappeared in *Tdo*-/- mice. The cytoarchitectural organization of *Tdo*-/- mice was the same as that of wild type mice. E, external plexiform layer; G, granule cell layer; Gm, glomerular layer; I, internal plexiform layer; LV, lateral ventricle; Mi, mitral cell layer; Mo, molecular layer; P, Purkinje cell layer; St, striatum; Su, subependymal zone.

It is well known that the SVZ is one of neurogenic regions in the mammalian CNS. Next, we addressed if TDO was expressed in the SVZ. As shown in Fig. 7A and 7B, we did not detect the immunoreactivity for TDO in the SVZ of either wild type or *Tdo*-/- mice. The TDO-immunoreactive structures in the olfactory bulb (OB), where neuroblasts derived from the SVZ migrate and differentiate to granule cells and periglomerular cells, were hardly observed, indicating that TDO may not be expressed in the SVZ-OB system. However, it cannot be excluded that TDO would be below detection level. Furthermore, we examined if another brain region, cerebellum, that contains granule cells express TDO. Granule cells in the cerebellum were immunoreactive for TDO, which is consistent with the relatively high expression of *tdo *mRNA in the cerebellum of the mice [12] (Fig. 7C). Further study to investigate the role of TDO in the cerebellum is needed. These results of TDO expression in the SVZ, the OB, and the cerebellum seem to be similar to those in the Allen Brain Atlas http://www.brain-map.org/, a database of gene expressions by in situ hybridization [19].

### Reduced TDO expression in the granule cells of CaMKIIα+/- mice

**Figure 8:**
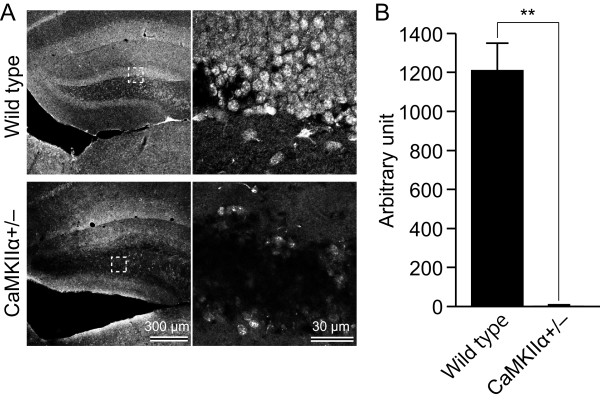
**Immunoreactivity of TDO in the granule cells of CaMKIIα+/- mice**. **A: **TDO was strongly expressed in the granule cells of wild-type mice, whereas TDO immunoreactivity was greatly reduced in CaMKIIα+/- mice, whose dentate gyrus (DG) granule cells display the physiologic and anatomic properties of immature cells. Note that interneurons in the hilus of both wild-type and CaMKIIα+/- mice expressed TDO, suggesting that the large decrease in TDO expression is specific to the DG granule cells. Higher magnification of the boxed-in area in each low-power image is displayed at the right of each lower-power image. **B: **Analysis of *tdo *expression with Real-time PCR. Total RNA was isolated from the DG of wild-type and CaMKIIα+/- mice. The expression of *tdo *mRNA in CaMKIIα+/- mice was significantly reduced to 0.39% of wild-type mice.

The findings described above indicate that TDO is expressed mainly in mature granule cells. We next examined the expression of TDO in mature cells using CaMKIIα+/- mice, in which most of DG granule cells do not express calbindin, and the electrophysiologic and morphologic features are strikingly similar to those of normal immature DG granule cells [11,20]. In the DG of wild-type littermate mice, TDO were expressed in the granule cells (Fig. 8A). In contrast, the expression of TDO was remarkably downregulated in the granule cells of CaMKIIα+/- mice (Fig. 8A). Importantly, because the expression level of TDO in the interneurons of the wild-type hilus was similar to those in CaMKIIα+/- mice, the decreased expression of TDO may be due to the lack of mature granule cells in CaMKIIα+/- mice.

We also quantified the expression of *tdo *mRNA using real-time PCR. The expression level of TDO in the DG of CaMKIIα+/- mice was 0.39% of that in the wild-type DG (Fig. 8B).

### Time course analysis with BrdU

**Figure 9:**
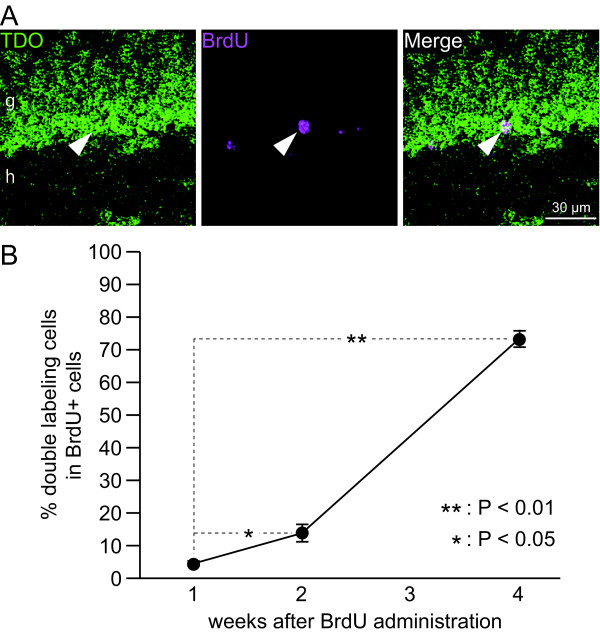
**Time-course of the double-labeling of TDO with bromodeoxyuridine (BrdU) in the dentate gyrus (DG) granule cells**. **A: **The images are representatives of double-labeling of BrdU and TDO at 4 wk after injection of BrdU. Arrowhead indicates the same cell in each line. g, granule cell layer; h, hilus. **B: **Changes in co-labeling rates of BrdU with TDO in the DG over a period of 4 wk after injection of BrdU are shown. Values are given as the means ± SEM of the analysis based on the results of three mice. The asterisk and double asterisk indicate statistically significant differences: *, p < 0.05; **, p < 0.01 (one-way ANOVA and Scheffé's post hoc test).

To determine when TDO was expressed during the development of granule cells, we performed BrdU labeling of granule cells. The rates of double-positive cells for TDO and BrdU were 4.47 ± 0.775, 13.8 ± 2.65, and 73.3 ± 2.52% at 1, 2, and 4 wk, respectively, after BrdU injection (Fig. 9). The number of TDO+/BrdU+ cells at 2 wk was significantly increased compared with that at 1 wk. TDO expression increased dramatically after 2 wk of age. Moreover, this result is consistent with the calretinin and calbindin staining results (Figs. 3 and 4) and a previous report that the appearance of calretinin begins 2 wk after the generation of granule cells and disappears by 4 wk, whereas granule cells begin to express calbindin at 4 wk [2,3,16,17]. Thus, these findings indicate that TDO begins to be expressed between 2 and 4 wk after the generation of granule cells.

### Spatial expression of TDO in the DG

**Figure 10:**
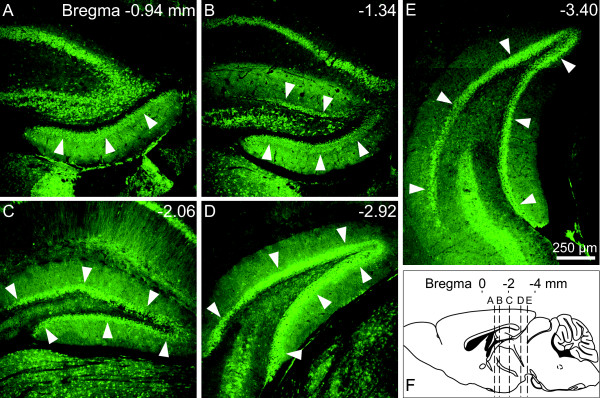
**Spatial expression of TDO in the dentate gyrus (DG)**. TDO immunoreactivity was almost uniformly detected along the anterior-posterior and dorsal-ventral axes. **A-E: **images of TDO between bregma -0.94 and -3.40. **F: **schematic representation of sagittal section. The positions of images A-E are indicated by dotted lines. Arrowheads indicate DG.

As described above, the immunohistochemical study showed that TDO was expressed in a part of immature granule cells and mature ones. We determined the spatial expression pattern of TDO in the DG. Almost uniform TDO immunoreactivity was detected along the anterior-posterior and dorsal-ventral axes (Fig. 10).

## Discussion

In the present study, we examined the spatial and temporal expression pattern of TDO in the granule cells of adult mouse DG. Using a combination of BrdU labeling and immunofluorescence staining with a specific anti-TDO antibody, we demonstrated that TDO expression began at the late-phase of granule cell development, around 2 to 4 wk after their generation. The mature granule cell marker calbindin was strongly co-expressed with TDO. In addition, in the DG of CaMKIIα+/- mice, in which granule cells remain immature [11], almost no TDO immunoreactivity was detected. These findings indicate that TDO is expressed mainly in mature granule cells and provide evidence that TDO is useful as a mature granule cell marker.

### TDO expression and development of newborn granule cells in the hippocampal DG

Adult neurogenesis is currently being intensively studied, in particular in the DG and the anterior subventricular zone. Although the temporal patterns of morphogenesis, electrophysiologic properties, and expression pattern of markers are gradually being elucidated [1-3], the mechanism of hippocampal adult neurogenesis remains largely unclear. In addition, the molecules identified in adult neurogenesis are not only developmental markers of newly generated neurons, but also play crucial roles in the development of granule cells. Thus, to elucidate the mechanism of adult neurogenesis, it is important to search for new molecular markers of developmental events of newborn granule cells. In the present study, we identified TDO as a novel mature granule cell marker. Since TDO was expressed in the mature granule cells of the dentate gyrus, it does not seem to play a role in the development of the dentate granule cells. In fact, the maturation of granule cells in the dentate gyrus of *Tdo*-/- mice is comparable to that of the control mice (unpublished results). In contrast, we previously have shown the increases in the numbers of neural stem cells and neural progenitor cells in the hippocampus of *Tdo*-/- mice, compared with *Tdo*+/+ mice [14]. This effect may be mediated by the increase in 5-HT. Chronic treatments with antidepressants, such as fluoxetine (FLX), a widely used selective serotonin reuptake inhibitor, stimulate the adult neurogenesis in the hippocampus [21,22]. Additionally, in the hippocampus of *Tdo*-/- mice, the level of 5-HT, which is synthesized from Trp, is 2-fold higher than that in control mice [14]. In addition, 5-HT is involved in the adult neurogenesis in the hippocampus [23]. Considering that the expression of TDO protein can be detected predominantly in the mature neurons, the rise in 5-HT may act on the neural stem cells/neural progenitor cells in *Tdo*-/- mice, and TDO may function in the mature granule cells as described below.

Within the central nervous system, TDO is the rate-limiting enzyme in the kynurenine pathway [13]. In this pathway, TDO begins to catabolize Trp to produce kynurenine, the downstream substrate of the pathway, which produces several neurotoxic and neuroprotective intermediates [13]. For example, quinolinic acid is excitotoxic, whereas kynurenine, kynurenic acid, and picolinic acid are neuroprotective [13]. In the hippocampus, kynurenine aminotransferase, the enzyme that produces kynurenic acid, is abundant in both neurons and astrocytes [24-26]. Furthermore, we demonstrated that mature DG granule cells strongly express TDO. Therefore, it is possible to speculate that TDO expressed in hippocampal mature neurons seems to be beneficial to protect from excitotoxicity by the synthesis of neuroprotective metabolites of the kynurenine pathway, such as kynurenine and kynurenic acid, which suppress N-methyl-D-aspartate (NMDA) and α7 nicotinic receptors in the hippocampus [26-28]. This consumption is further supported by the notion that the kynurenine, downstream metabolite of TDO, is capable of inducing expression of the neuronal survival factor, nerve growth factor [29-31] and the kynurenic acid is capable of mediating suppression of excessive NMDA and α7 nicotinic receptor activity [27,28]. Further examination of the role of TDO in neuroprotection is needed.

Several molecular markers and their expression time course have been identified in hippocampal adult neurogenesis: GFAP for type 1 progenitors; DCX and PSA-NCAM for type 2 and 3 progenitors and immature neurons 1 to 2 wk of age; calretinin for immature neurons 2 to 3 wk of age, when new granule cells send out their axons to CA3 [32,33]; and calbindin for mature neurons [2,3]. In the present study, we identified TDO as a new molecular marker of mature granule cells in the hippocampus. A mature granule cell marker, calbindin, is neuroprotective against glutamate excitotoxicity [34,35]. Calbindin is an EF-hand Ca^2+ ^binding protein that regulates intracellular Ca^2+ ^homeostasis [34,35]. The expression of calbindin begins at 4 wk after granule cell generation [2,3]. This period correlates with near completion of the excitatory and inhibitory synapses on granule cells. On the other hand, TDO is expressed beginning at 2 to 3 wk after granule cell generation, when granule cells begin to receive synaptic inputs from the entorhinal cortex [2,3]. Furthermore, considering the postulated function of TDO described above, TDO might also be associated with the acquisition and maintenance of granule cell functions, such as synapse formation and maintenance, and neuroprotection.

### Usefulness of TDO as a maturation marker of the dentate granule cells

Calbindin, which is generally used as a mature granule cell marker, widely and strongly expresses in the hippocampal formation, such as CA regions and interneurons. Thus, in the DNA array or real-time PCR analysis of whole hippocampus of CaMKIIα+/- mice, calbindin expression was about 60% of its level in control mice, whereas the ratio of TDO to its control level was only 10% [36], suggesting that TDO may be more sensitive mature granule cell marker in the hippocampal adult neurogenesis. Likewise, in our most recent paper that reported the reversal of hippocampal neuronal maturation by serotonergic antidepressants, FLX, mRNA expression of *tdo* in the dentate gyrus of FLX-treated mice was less than 20% of its level in control mice, whereas in the case of calbindin the ratio to the control is 35% [37]. These results also indicate that TDO may be a useful marker for granule cell maturation, especially when whole hippocampal sample is used for quantitative PCR and Western blot analyses.

### Implication of TDO on the function of the dentate granule cell

TDO is closely related to psychiatric disorders, such as schizophrenia, depression, and bipolar disorder [38-40]. Interestingly, abnormal up-regulation or down-regulation of the kynurenine pathway has been reported in patients with psychiatric disorders. For example, in the neocortex of patients with schizophrenia and bipolar disorder, kynurenine and kynurenic acid levels are significantly increased [39,41]. In contrast, the plasma levels of both kynurenine and kynurenic acid are significantly reduced in depression [40]. In the kynurenine pathway, kynurenic acid controls sensory gating by regulating the activity of α7 nicotinic and NMDA receptors [42-45]. Additionally, 5-HT is synthesized from Trp [13], suggesting that the reduced expression of TDO may lead to an increase in 5-HT synthesis [14]. Importantly, we recently reported that FLX treatment, which increases in the concentration of 5-HT in the brain parenchyma, induces "dematuraion" of the dentate granule cells [37]. TDO may also regulate an array of behaviors including sensorimotor control, cognition, and mood via the control of 5-HT content [46]. These data suggest that TDO may play an important role in the cellular state and function of the dentate granule cells.

## Methods

### Animals

For immunohistochemical studies, 8-wk-old male C57BL/6J mice (4 mice for each time point, control, 1, 2, and 4 wk after BrdU administration; total 16 mice; Charles River Laboratories International Japan Inc., Shiga, Japan) were maintained under a normal light-dark cycle (12 h light/12 h dark). Animals were injected intraperitoneally with BrdU (50 mg/kg body weight) every 24 h over 5 d to label newborn neurons. At 1, 2, and 4 wk after the last BrdU injection, animals were deeply anesthetized and transcardially perfused with 4% paraformaldehyde in 0.1 M phosphate buffer, pH 7.4. The brains were dissected, immersed overnight in the same fixative, and transferred to 30% sucrose in phosphate buffered saline (PBS) for at least 3 d for cryoprotection. We used CaMKIIα+/- mice obtained from Jackson Laboratories (Bar Harbor, ME), which were also utilized in our previous study [11]. Then, heterozygous mice were crossed with C57BL/6 mice for at least 16 generations. We used heterozygous CaMKIIα knockout mice, because it is difficult to obtain homozygotes due to mating problems between heterozygous male and heterozygous female mice. *Tdo*-/- mice [14] were also used to check the specificity of the anti-TDO antibody [14]. Mice were housed and perfused using the same methods (for CaMKIIα heterozygous mice, 6 wild-type and 6 heterozygous mice; for *Tdo*-/- mice, 6 wild-type and 5 knockout mice; all 8-wk-old males). The brain samples were stored at 4°C until use. For Western blot analysis, three 8-wk-old male wild-type and 3 *Tdo*-/- mice were used. All animal experiments were approved by the Institutional Animal Care and Use Committee of Fujita Health University, based on Law for the Humane Treatment and Management of Animals (2005) and Standards Relating to the Care and Management of Laboratory Animals and Relief of Pain (2006) or approved by the Institutional Animal Care and USE Committee of Osaka University Graduate School of Medicine. Every effort was made to minimize the number of animals used.

### Antibody characterization

In this study, we used the following antibodies as primary antibodies: a mouse polyclonal antibody for TDO; mouse monoclonal antibodies for β-tubulin, GFAP, Ki-67; rabbit polyclonal antibodies for calretinin, calbindin, and gamma-aminobutyric acid (GABA); a rat monoclonal antibody for BrdU; and a guinea pig polyclonal antibody for DCX.

The molecular weight of TDO is 45 kDa [47,48]. With the anti-TDO antibody (Abnova, Corp., Taipei, Taiwan: Lot. Number 08234 WULZ), a 45-kDa band that appeared to be TDO and an 18-kDa band were observed (Fig. 1A, lane 1) [48]. In lane 2, which contained total protein from *Tdo*-/- mice, an 18-kDa band was detected, suggesting that the secondary antibody labeled an extra common band. As expected, when the Western blot analysis was performed without the primary antibodies, the same band appeared in the negative control (lane 3). In addition, we determined the specificity of the anti-TDO antibody in mouse brain tissues. In wild-type mice, the antibody visualized the granule cells of the DG, interneurons in the hilus region, CA3, and CA1 neurons (Fig. 1B). On the other hand, in the *Tdo*-/- mice, immunoreactivity was not observed in the granule cell layer, CA3, and CA1. We concluded that anti-TDO antibody was specifically reactive for TDO protein. Caution that Lot. Number is important for the specificity of anti-TDO antibody and thus the specificity of anti-TDO antibody should be re-examined if the different Lot. anti-TDO antibody is used.

Mouse monoclonal anti-β-tubulin (clone TUB2.1, Sigma-Aldrich, St. Louis, MO, T4026) was produced by immunization with purified rat brain tubulin, and reacts with human, bovine, rat, mouse, and chick tissue. It recognizes a 55-kDa protein in Western blot analysis (manufacturer's technical information). The specificity of this antibody was confirmed in a previous study [49].

The rabbit polyclonal anti-GFAP antibody (Sigma-Aldrich, G3893) was generated against GFAP isolated from cow spinal cord. The antibody is specific to astrocytes and ependymal cells of the central nervous system (manufacturer's technical information). The specificity of this antibody was confirmed in a previous study [50].

The antibody directed against Ki-67 was generated with recombinant human Ki-67 protein as the immunogen (BD Pharmingen, Franklin Lakes, NJ, 550609). This antibody recognizes two bands (345 and 395 kDa) on Western blot analyses of human cells, consistent with the molecular weights of alternatively spliced Ki67 isoforms [51]. The anti-Ki67 antibody labels neuronal precursor cells and stem cells in the hippocampal DG [52].

A guinea pig polyclonal anti-DCX antibody raised against a synthetic peptide comprising amino acids 350-365 of mouse DCX (Chemicon, Temecula, CA, AB5910) was used in this study. On Western blots, this antibody recognizes a single 40-kDa band, which corresponds to the calculated molecular mass of DCX [53]. Staining obtained with this antibody is identical to that described for other anti-DCX antibodies [9]. DCX labeling was prominent in the DG and DCX-labeled cells outside neurogenic regions of the brain were only rarely detected [9].

Rabbit polyclonal anti-calretinin antibody (Chemicon, AB5054) was prepared against a recombinant of the entire amino acid sequence of rat calretinin. This antibody recognizes calretinin in human, rat, and mouse (manufacturer's technical information). This antibody recognizes both calcium-bound and calcium-unbound conformations of calretinin by Western blot (32 kDa; manufacturer's technical information). In addition, the distribution of calretinin neurons in the hippocampi of our processed tissue was identical to that in previous reports [54,55].

Rabbit polyclonal anti-calbindin antibody (Swant, Bellinzona, Switzerland, CB 38) was raised against recombinant rat calbindin D-28k, according to information provided by the manufacturer, and is specific for calbindin in Western blot [56]. Immunohistochemical staining is abolished by preadsorption with calbindin protein [57], and this antibody labels granule cells in the adult DG [58].

Rabbit polyclonal GABA antiserum (Sigma-Aldrich, A2052) was prepared against GABA conjugated to bovine serum albumin (BSA). This antibody shows positive binding with GABA, and negative binding with BSA in a dot blot assay (manufacturer's technical information). The pattern of GABA labeling we observed in the DG was identical with that described previously [59].

Monoclonal rat anti-BrdU antibody (Abcam, Cambridge, UK, ab6326) reacted to the nuclei of cells that had incorporated BrdU during S-phase of the cell cycle. We observed a pattern of staining identical with that described in previous reports of studies using the rat antibody [60-62].

Monoclonal rabbit anti-neuronal class III beta-tubulin (Tuj1) antibody (Covance, Emeryville, CA, MRB-435P) is highly reactive to neuron specific class III beta-tubulin. This antibody does not identify beta-tubulin of glial cells (manufacturer's technical information). We observed a pattern of staining identical with that described in previous report using the antibody [63].

Polyclonal rabbit anti-Iba1 antibody (Wako, Osaka, Japan, 019-19741) is raised against a synthetic peptide corresponding to C-terminal of Iba1. This antibody is reactive to microglia and macrophage, but not to neurons and astrocytes (manufacturer's technical information). The immunohistological pattern of Iba1 reactive cells in the hippocampi of our processed tissue was identical to that in previous report [64].

Polyclonal rabbit anti-olig2 antibody (IBL, Gunma, Japan, 18953) is raised against a synthetic peptide of C-terminal of human olig2. The immunohistological pattern of olig2 reactive cells in the hippocampi of our processed tissue was identical to that in previous report [65].

### Western blot analysis

Mice were deeply anesthetized and their brains were removed and immersed in ice-cold PBS. The hippocampus was dissected out and stored at -80°C until use. Tissues in five volumes of PBS containing 0.1% Triton X-100, 1 μg/ml leupeptin, 1 μg/ml aprotinin, and 1 mM phenylmethylsulphonyl fluoride were sheared into small pieces with a pair of scissors, lysed with microhomogenizers, and stored on ice for 5 min. The resulting solutions were centrifuged at 15,000 × g for 30 min at 4°C. The protein content of the supernatants was determined by the Lowry method. The supernatants were boiled for 3 min in sodium dodecyl sulfate (SDS) sample buffer (62.5 mM Tris-HCl, 10% glycerol, 2% SDS, 0.05% bromophenol blue, and 1% 2-mercaptoethanol) and centrifuged at 5000 × g for 1 min at 4°C. The supernatants were stored at -80°C until use.

Aliquots of the supernatants (20 μg total protein) were loaded on 4% to 12% SDS polyacrylamide gradient gels, and transferred to polyvinylidene difluoride membranes (GE Healthcare, Buckinghamshire, England). Membranes were immersed in 5% skim milk (Morinaga, Tokyo, Japan) in PBS containing 0.005% Tween-20 (PBST) at room temperature for 1 h with gentle agitation. They were incubated at 4°C overnight with anti-TDO antibody diluted 1:200 and anti-β-tubulin antibody diluted 1:200 with 5% skim milk solution. Membranes were washed 3 × 10 min in PBST and incubated with a secondary antibody conjugated with horseradish peroxidase (Santa Cruz Biotechnology, Santa Cruz, CA) diluted 1:1000 with 5% skim milk solution at room temperature for 1 h. Following the 3 × 10 min washes with PBST, proteins were detected with the enhanced chemiluminescence (ECL System, GE Healthcare) and a cooled CCD camera Light-Capture (ATTO, Tokyo, Japan).

### Histology

Brains were mounted in Tissue-Tek (Miles, Elkhart, IN), frozen, and cut into 50-μm thick coronal sections using a microtome (CM1850, Leica Microsystems, Wetzlar, Germany). Sections were stored in PBS containing sodium azide (0.05%, w/v) at 4°C until staining.

For immunostaining of TDO, sections were incubated at 80°C for 30 min in 10 mM citrate buffer, pH 9.0, to retrieve and enhance their antigenicities and staining intensities. When double-staining with BrdU was performed, the sections were further incubated at 4°C for 10 min in 0.1 N HCl and then at 37°C for 30 min in 2 N HCl. Sections were washed for 30 min in PBS, and then blocked in 0.2 M glycine in PBS at room temperature for 2 h. After washing in PBS for 1 h, sections were preincubated with PBS-DB (4% normal donkey serum [Vector Laboratories, Burlingame, CA] and 1% BSA in PBS) for 2 h at room temperature. The sections were incubated at 4°C for 48 h or at room temperature for 12 h with antibodies. We used the following primary antibodies: mouse polyclonal anti-TDO (1:500); mouse monoclonal anti-GFAP (1:200) and anti-Ki-67 (1:20); rat monoclonal anti-BrdU (1:100); rabbit monoclonal anti-Tuj1 (1:1,000); rabbit polyclonal anti-GABA (1:2,000), anti-calretinin (1:3,000), anti-calbindin (1:5,000), anti-Iba1 (1:200), anti-olig2 (1:200); and guinea pig polyclonal anti-DCX (1:1,000). After washing in PBS for 1 h, the sections were incubated at room temperature for 1 h with the following secondary antibodies: anti-mouse IgG Alexa 488 (1:200; Molecular Probes, Eugene, OR), anti-rat IgG Alexa 594 (1:200; Molecular Probes), and anti-rabbit IgG Alexa 546 (1:200; Molecular Probes). After washing in PBS for 1 h, the sections were mounted on glass slides coated with 3-aminopropyltriethoxysilane and embedded with Permafluor (Thermo Shandon, Pittsburgh, PA). We used a confocal microscope (LSM 510 META; Zeiss, Göttingen, Germany) to obtain images of the stained sections.

### Quantitative RT-PCR

RT-PCR was performed according to our previous study [11]. Briefly, total RNA was isolated from the DG of 27 to 29-wk-old wild-type mice and CaMKIIα mice. First-strand cDNA was prepared from 2 μg of DNase I-treated total RNA using SuperScript III reverse transcriptase (Invitrogen, Carlsbad, CA). The expression of related genes was quantified using the SYBR green reagent (2× SYBR Green PCR Master Mix; Qiagen, Valencia, CA) following the instructions of the manufacturer. Quantitative PCR was performed using DNA Engine Opticon 2 Real-Time PCR Detection System (Bio-Rad, Hercules, CA) with the following conditions: 15 min at 95°C, then 45 cycles of 15 s at 94°C, 30 s at 60°C, 30 s at 72°C and 1 min at 65°C. β-Actin was amplified from all samples to normalize expression. The following primers were used: TDO (1-105), 5'-ATGAGTGGGTGCCCGTTTG and 5'-GGCTCTGTTTACACCAGTTTGAG; β-actin (851-962), 5'-AGTGTGACGTTGACATCCGTA and 5'-GCCAGAGCAGTAATCTCCTTCT. Ct values used were the means of duplicate replicates.

### Quantification

Analysis was performed using a confocal microscope equipped with a 40× objective (Plan-NEOFLUAR, NA = 0.75, Zeiss) and a pinhole setting that corresponded to less than 1-μm thickness of the focal plane. To exclude false positives due to the overlay of signals from different cells, randomly selected positive cells were analyzed by moving through the entire z-axis of each cell. Cells were counted under the live mode of confocal scanning. A minimum of 100 BrdU-, calretinin-, GFAP-, DCX-, and Ki-67-positive cells was examined for co-labeling with TDO in each animal and at each time point. Data are presented as the mean percentage of BrdU+ cells co-labeled with TDO (mean ± SEM). The quantification of double-positive cells was analyzed with a two-way ANOVA. If a significant main effect was detected by ANOVA, a Tukey's post hoc test was used to determine the source of the detected significance in the ANOVA.

## Competing interests

The authors declare that they have no competing interests.

## Authors' contributions

TM directed all experiments and wrote the manuscript. KO participated in the design of the study, carried out the immunohistological and western blot experiments, and co-wrote the manuscript. HH, K Toyama, and K Takao performed the dissection of mouse brains and quantitative RT-PCR. MK, HF, and TN developed TDO knockout mice. All authors read and approved the final manuscript.
